# Low triglyceride levels are associated with a better metabolic control in patients with type 1 diabetes

**DOI:** 10.1186/1758-5996-3-22

**Published:** 2011-09-02

**Authors:** Leticia M Alcantara, Nathalia E Silveira, Joana R Dantas, Paula B Araujo, Marcus M de Oliveira, Adolpho Milech, Lenita Zajdenverg, Melanie Rodacki, José EP de Oliveira

**Affiliations:** 1 Nutrology Section, Hospital Universitario Clementino Fraga Filho - Universidade Federal do Rio de Janeiro (UFRJ), Rio de Janeiro/RJ, Brazil

## Abstract

**Background:**

Although it is well known in the literature that high triglyceride serum (TG) levels can jeopardize the metabolic control, little is known about the influence of low TG on type 1 diabetes patients (T1D). The aim of this study is to investigate the distribution of TG serum levels in individuals with T1D and its relationship with metabolic control.

**Findings:**

We reviewed the medical charts of 180 patients with T1D, who were classified in groups according to TG levels: 1) low (below 50 mg/dL); 2) normal (50-150 mg/dL); 3) high (above 150 mg/dL). TG were low in 21.1% (n = 38; group 1), normal in 68.6% (n = 123; group 2) and high in 10.6% (n = 19; group 3). High TG was associated with a poor metabolic control (p < 0.001). Patients with TG lower than 50 mg/dL had a lower HbA1c than those with TG between 50 and 150 mg/dL (7.41+/-1.50% vs 8.56%+/-1.94%; p = 0.002).

**Conclusion:**

TG lower than 50 mg/dL was common and might be associated with a better metabolic control in patients with T1D, although it is not clear whether the former is the cause or consequence for the latter.

## Introduction

Type 1 diabetes (T1D) is a chronic autoimmune disease characterized by insulin deficiency (1). Although it is not usually associated with obesity, insulin resistance and hypertriglyceridemia, it is known that the increase in the worldwide prevalence of obesity and dyslipidemia is also affecting individuals with T1D (2). The relationship between cardiovascular disease and hypertriglyceridemia (3) has been well established, both in type 1 diabetes (4) and type 2 diabetes (5). Insulin influences the activity of lipase lipoprotein and therefore insulin deficiency and poor metabolic control are associated with hypertriglyceridemia (6). Although much is said about hypertriglyceridemia and its importance, little is known about the relevance of low triglyceride levels in the general population or in those with T1D. However, hypotriglyceridemia has been linked to autoimmune diseases (7) and may be implicated in the development of T1D in individuals at risk for the disease (8). The aim of this study was to investigate the distribution of triglyceride (TG) serum levels in individuals with T1D and its possible clinical implications.

## Research design and methods

We performed a retrospective analysis of the medical charts of patients with T1D at the Hospital Universitario Clementino Fraga Filho, in Rio de Janeiro. All patients signed an informed consent and the protocol was approved by the Institutional Review Board. Clinical data, cholesterol, triglycerides, serum glucose and glycated hemoglobin (HbA1c) were retrieved. Patients were classified in three groups according to their TG levels: 1) low (< 50 mg/dL); 2) normal (50 to 150 mg/dL) and 3) high TG (more than 150 mg/dL). None of patients included were engaged in intense physical activity. Chi Square test, Mann Whitney and ANOVA Kruskall Wallis were used to compare groups and Spearman coefficient was used for correlation tests. An alpha < 0.05 was employed and all tests were two-tailed. SPSS 13.0 for Windows was used for statistical analysis.

## Results

The population comprised 180 individuals (100 women, 80 men). Their mean age, disease duration and age at onset were 25.2 ± 8.4, 12.6 ± 7.3 and 12.7 ± 8.3 years. Their mean insulin dose/kg of body weight ratio was 0.95 ± 0.42 U/kg and their mean body mass index (BMI) was 23.9 ± 3.2 kg/m^2^. Retinopathy was found in 15.6% and nephropathy in 17.8% (mild in all cases). 34% were overweight and 6.4% were obese. Their mean fasting glucose and HbA1c were 173.8 ± 102.9 mg/dL and 8.6 ± 2.2%. The mean serum cholesterol, HDL, LDL and TG were 166.6 ± 39.8 mg/dL, 51.6 ± 15.6 mg/dL, 92.2 ± 29.3 mg/dL and 95.7 ± 76.8 mg/dL. High LDL levels was seen in 35.2% (100-130 mg/dL in 78.7%, > 160 mg/dL in 9.8% of these); 20% used statins (58.3% of these had LDL ≥ 100 mg/dL; > 130 mg/dL in only 6 cases). TSH was normal in 95% and 5% had subclinical hypothyroidism. Liver diseases were not observed.

**Figure 1:**
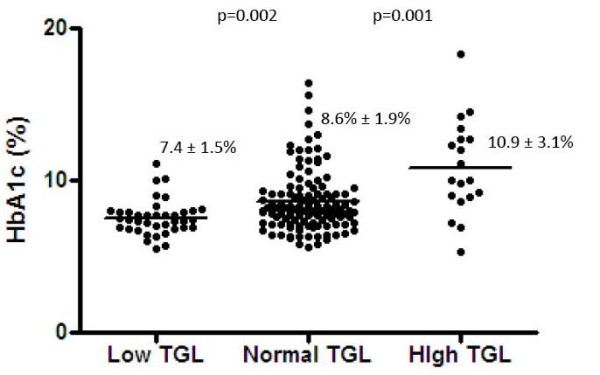
**HbA1c according to the groups with low, normal and high triglyceride**. TGL: triglyceride level.

TG levels were low in 21.1% (n = 38; group 1), normal in 68.3% (n = 123; group 2) and high in 10.6% (n = 19; group 3). Clinical parameters according to the TG levels are described in the table 1. HbA_1c _differed between groups (p = 0.039). The difference was seen not only between groups 3 and others (10.88 ± 3.1% vs 8.3 ± 1.9%; p < 0.001) but also between groups 1 and 2 (7.41 ± 1.50% vs 8.56% ± 1.94%; p = 0.002), as shown in Figure 1. There was a direct correlation between TG and HbA1c in the whole sample (R = 0,34; p < 0.001) and also in those with TG < 150 mg/dL (R = 0.25; p = 0.002). The frequency of individuals with HbA1c < 7% differed between groups 1 and 2 (34.21% vs 17.88%; p = 0.025). A trend towards a lower insulin dose/kg was also seen in group 1 when compared to group 2 (0.84 ± 0.30 U/kg vs 1.00 ± 0.46 U/kg; p = 0.052), but not between those and group 3 (0.84 ± 0.31 U/kg; p = 0.31).

**Table 1:** Clinical parameters according to the TG levels

	Low TG	Normal TG	High TG
Frequency (%)	21.1	68.3	10.6
Age of diagnosis (years)	23.49 (± 6.45)	25.81 (± 9.15)	25.00 (± 7.17)
Duration of disease (years)	11.84 (± 5.47)	12.79 (± 7.3)	13.00 (± 6.7)
BMI (Kg/m^2^)	22.58 (± 2.50)	24.33 (± 3.25)	24.37 (± 3.63)
Overweight* (%)	12.2	34.2	42.1
Obesity **(%)	4.9	5.8	15.8
Daily insulin dose (IU/kg)	0.83 (± 0.30)	1.01 (± 0.47)	0.84 (± 0.31)
Fasting glucose (mg/dL)	143.37 (± 89.68)	180.11 (± 95.86)	196.24 (± 154.76)
A1 hemoglobin (%)	7.67 (± 1.50)	8.56 (± 1.95)	10.85 (± 1.95)
Total cholesterol (mg/dL)	144.98 (± 24.68)	166.08 (± 28.35)	216.37 (± 73.43)
LDL (mg/dL)	77.67 (± 22.16)	93.97 (± 25.51)	112.89 (± 49.02)
HDL (mg/dL)	54.0 (± 14.43)	51.14 (± 15.25)	49.31 (± 19.95)
TG (mg/dL)	42.65 (± 5.93)	85.25 (± 26.70)	276.16 (± 11.21)

Table legend: TG = triglycerides; BMI = body mass index; LDL = low density lipoprotein; HDL = high density lipoprotein; *overweight = BMI≥25 Kg/m^2 ^, **obesity = 30 Kg/m^2 ^.

There was a significant difference in BMI between groups (22.5 ± 2.5 kg/m^2 ^24.3 ± 3.3 kg/m^2 ^and 24.3 ± 3.6 kg/m^2 ^in groups 1, 2 and 3; p = 0.018). This difference was seen only between groups 1 and 2 (p = 0.002), and not between those and group 3 (p = 0.986). There was no correlation between BMI and HbA1c (R = 0.038; p = 0.64). A significant difference in LDL levels was seen between groups 1 and 2 (76.9 ± 22.5 vs 93.8 ± 25.3; p < 0.0001) but not between 2 and 3 (112.9 ± 49; p = 0.076).

## Discussion

The relationship of TG levels and glycemic control has been recognized for a long time, as well as their association with cardiovascular mortality when they are above normal levels (4,5). However, this study suggests that not only high TG levels are associated with poor glycemic control, but that low TG might also have clinical implications.

We found hypertriglyceridemia in 10.6% of patients with T1D, which is similar to the frequency described in other populations (4). In our study, the group with higher TG levels had a higher BMI, higher A1C and used a similar insulin dose than others. This is probably associated with worse dietary habits in this group. Moreover, our data indicates that TG < 50 mg/dL are associated with a more favorable metabolic profile such as lower HbA1c, BMI, cholesterol and, possibly, insulin dose. Whether the association between low TG and glycemic control is a cause or a consequence remains to be elucidated. It is possible that individuals that tend to have lower TG levels also present genetic (9) or environmental factors that lead them to a better metabolic profile. On the other hand, the decrease in TG levels might only be a consequence of a good glycemic control or the habit of a low-carbohydrate diet to prevent glycemic excursions (10). None of the individuals had thyroid diseases that could explain the low TG levels or were engaged in intense physical activity (11,12). Interestingly, it has been suggested that individuals with autoimmune disease have lower TG levels (7) and a significant percentage of our sample exhibited TG < 50 mg/dL. It is possible that the cut-off for high TG levels in these patients, including those with T1D, should be lower than the usual parameters. If this is true, the explanation for our data might be that part of the values that are considered in the normal range should be actually considered high for this population with autoimmune disease. It is possible that by establishing a lower cut-off for high triglycerides in these patients, no further differences would be seen between individuals with low and normal TG. Studies considering the cardiovascular outcome are still lacking in lower TG levels, which was not addressed in this manuscript. One limitation of our study is the lack of a control group. As the study group is comprised by young and mostly lean individuals, the presence of a high frequency of patients with TG < 50 mg/dL might be a characteristic of this age group, independently of their disease. Although to our knowledge the frequency of TG < 50 mg/dL in healthy young individuals has not been reported, a higher mean of TG levels was previously identified in young adults when compared to our data (13). To conclude, TG < 50 mg/dL are common in patients with T1D in our population and might be associated with a better metabolic control. However, it is not clear whether the former is the cause or the consequence for the latter.

## Competing interests

The authors declare that they have no competing interests.

## Authors' contributions

LMA and NES equally participated in the data collection and drafted the manuscript. JRD performed the statistical analysis and reviewed the manuscript typography (translation to English language). PBA participated in the data collection. MMO participated in the data collection and in the study design. AM participated in the coordination of the study. LZ participated in the study design and performed the final corrections. MR conceived the study, performed the statistical analysis and also made the final corrections. JEPO participated in the study coordination. All contributors read and approved the final manuscript.
